# Antimicrobial resistant bacteria recovered from retail ground meat products in the US include a *Raoultella ornithinolytica* co-harboring *bla*_KPC-2_ and *bla*_NDM-5_

**DOI:** 10.1038/s41598-021-93362-x

**Published:** 2021-07-07

**Authors:** Gregory A. Ballash, Amy L. Albers, Dixie F. Mollenkopf, Emily Sechrist, Rachael J. Adams, Thomas E. Wittum

**Affiliations:** College of Veterinary Medicine, Department of Veterinary Preventive Medicine, The Ohio State University, Columbus, OH USA

**Keywords:** Antimicrobial resistance, Infectious diseases, Public health

## Abstract

Retail beef and pork, including processed products, can serve as vehicles for the zoonotic foodborne transmission of pathogens and antimicrobial resistant bacteria. However, processed and seasoned products like sausages, are not often included in research and surveillance programs. The objective of this study was to investigate retail ground beef and pork, including processed products, for the presence of common foodborne pathogens and antimicrobial resistant bacteria. We purchased 763 packages of fresh and fully cooked retail meat products during 29 visits to 17 grocery stores representing seven major grocery chains located in west and central Ohio. Each package of meat was evaluated for contamination with methicillin-resistant *Staphylococcus aureus* (MRSA), *Salmonella* spp., *Enterobacteriaceae* expressing extended-spectrum cephalosporin resistance, and carbapenemase-producing organisms (CPO). Only 3 of the 144 (2.1%) packages of fully cooked meat products contained any of these organisms, 1 with an extended-spectrum β-lactamase-producing (ESBL) *Enterobacteriaceae* and 2 with CPO. Among the 619 fresh meat products, we found that 85 (13.7%) packages were contaminated with MRSA, 19 (3.1%) with *Salmonella,* 136 (22.0%) with *Enterobacteriaceae* expressing an AmpC (*bla*_CMY_) resistance genotype, 25 (4.0%) with *Enterobacteriaceae* expressing an ESBL (*bla*_CTX-M_) resistance genotype, and 31 (5.0%) with CPO, primarily environmental organisms expressing intrinsic carbapenem resistance. However, one CPO, a *Raoultella ornithinolytica*, isolated from pork sausage co-harbored both *bla*_KPC-2_ and *bla*_NDM-5_ on IncN and IncX3 plasmids, respectively. Our findings suggest that fresh retail meat, including processed products can be important vehicles for the transmission of foodborne pathogens and antimicrobial resistant bacteria, including those with epidemic carbapenemase-producing genotypes.

## Introduction

Foodborne diseases cause an estimated annual burden of 600 million illnesses and 420,000 deaths globally^1^. In the US alone, there are an estimated 48 million cases of foodborne illness and approximately 3,000 foodborne-related deaths per year^2,3^. Bacteria are important foodborne pathogens, causing approximately 9% of the total foodborne illnesses annually^4^. Among the bacterial pathogens causing foodborne illness, nontyphoidal *Salmonella* spp*., Staphylococcus aureus*, *Enterobacteriaceae* including *Escherichia coli,* and *Campylobacter jejuni* are common zoonotic foodborne pathogens, causing approximately 40% of the bacterial attributed foodborne illnesses^3^.

Retail ground pork and beef products are frequently contaminated with foodborne pathogens and serve as transmission vectors for these pathogens via raw or undercooked meat or by cross contamination^5,6^. Together beef and pork sources account for 12% of the yearly estimated foodborne illnesses and rank among the top 10 sources for zoonotic foodborne outbreaks, illness, and hospitalizations^4^. Moreover, both beef and pork can be reservoirs for antimicrobial resistant (AMR) bacteria and mobile genetic elements (MGE) that confer resistance to medically important antibiotics^5–7^.

The US maintains national surveillance programs to monitor foodborne pathogens and antimicrobial resistant bacteria in meat products^8^. However, these programs cannot possibly evaluate all varieties of meat products and often choose those that are most popular among consumers. Processed raw meat products—defined as products that include additives like salt, spices and preservatives—are not often sampled as part of these surveillance programs, but they account for approximately 40% of all red meat consumption in the US^9^. More concerning is that carbapenemase-producing organisms (CPO) and their resistance genes have been isolated from both cattle and swine, suggesting a new emerging threat to the retail meat food chain^10,11^. Here we aim to survey popular retail ground pork and beef consumer items, including processed products, to estimate their frequency of contamination with foodborne pathogens and AMR bacteria including MRSA, *Salmonella*, extended-spectrum cephalosporin resistant *Enterobacteriaceae,* and carbapenem-resistant Enterobacterales. Additionally, we assess the association between the recovery of these pathogens and potential risk factors including product type and processing characteristics.

## Results

**Figure 1:**
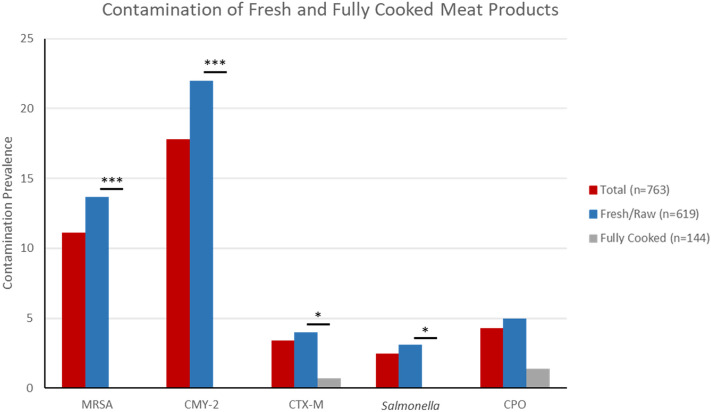
Total contamination of retail meat products by each of the bacterial contaminants (MRSA, AmpC and ESBL-producing *Enterobacteriaceae*, *Salmonella,* and carbapenemase-producing organisms). The total contamination was stratified by the cooked status of the meat products sampled (Fresh vs. Fully Cooked). Asterisks represent *P*-values for comparing bacterial contamination vs. cooked status (* = 0.05 > *P* > 0.01, *** = 0.001 > *P*).

We purchased a total of 763 packages of retail meat products for inclusion in this study. Of these, 85 (11.1%) harbored MRSA, 19 (2.5%) were contaminated with nontyphoidal *Salmonella*, 136 (17.8%) contained *Enterobacteriaceae* expressing an AmpC phenotype conferred by *bla*_CMY_, 27 (3.5%) contained *Enterobacteriaceae* expressing an ESBL phenotype encoded by *bla*_CTX-M_, and 33 (4.3%) harbored CPO (Fig. 1). There were 144 (18.9%) fully cooked and 619 (81.1%) fresh meat products (Fig. 1). Among the fully cooked products, one package (0.7%) contained *Enterobacteriaceae* expressing an ESBL phenotype and harboring *bla*_CTX-M-15_, and two packages (1.4%) harbored a CPO, both identified as *Pseudomonas* spp. Among the fresh meat products, 85 packages (13.7%) were contaminated with MRSA, 19 (3.1%) with *Salmonella*, 136 (22.0%) with *Enterobacteriaceae* expressing an AmpC (*bla*_CMY_) genotype, 25 (4.0%) with *Enterobacteriaceae* expressing an ESBL (*bla*_CTX-M_) genotype and 31 (5.0%) with CPO, primarily environmental organisms expressing intrinsic and/or chromosomally-mediated carbapenem-resistance.

**Figure 2:**
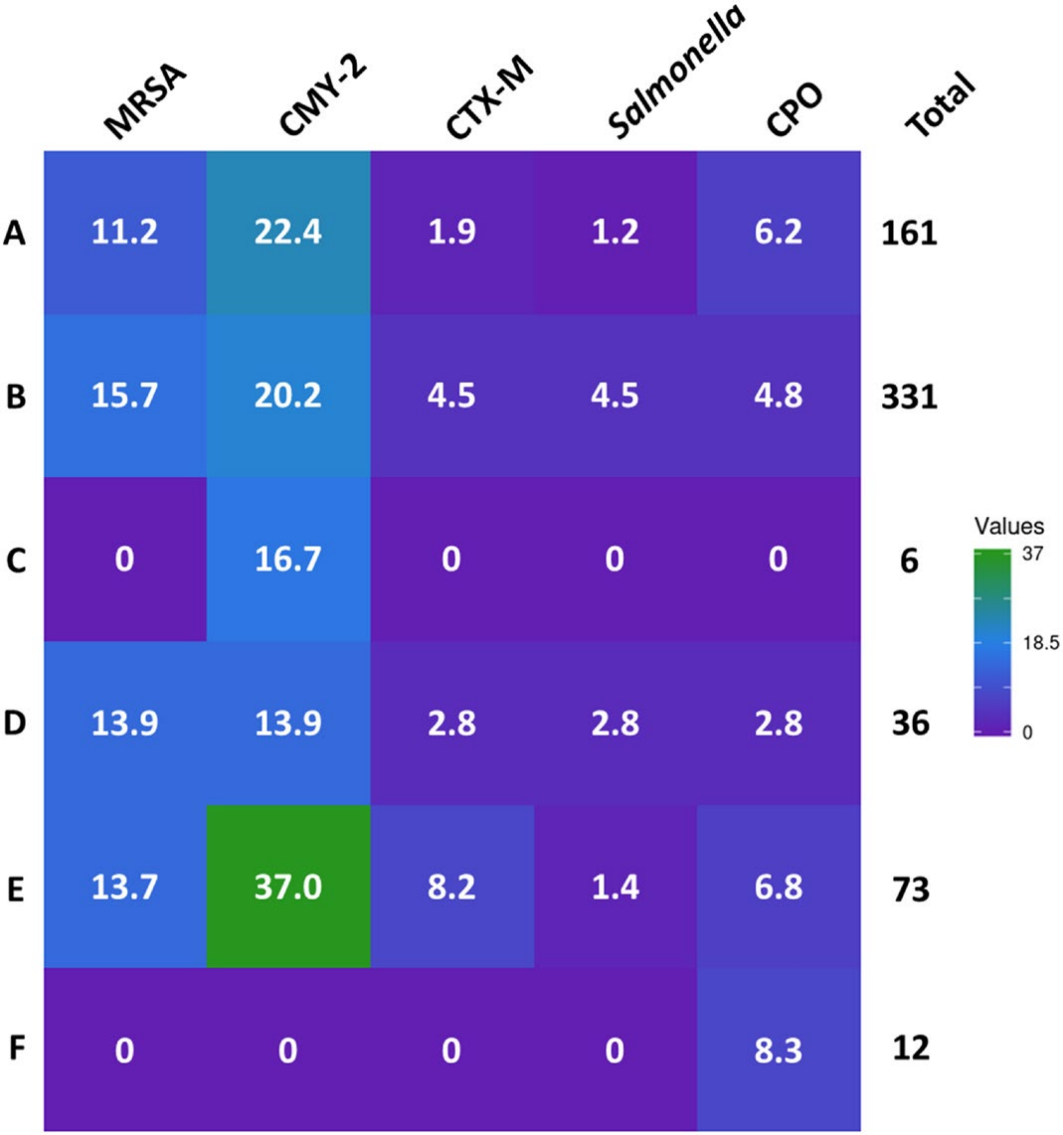
Pairwise heatmap of contamination prevalence with each of the bacterial contaminants isolated from each of the grocery chains labeled A–F. The total number of meat samples collected from each of the grocery stores is along the right vertical axis of the heatmap. The heatmap was visualized using Heatmapper.

**Figure 3:**
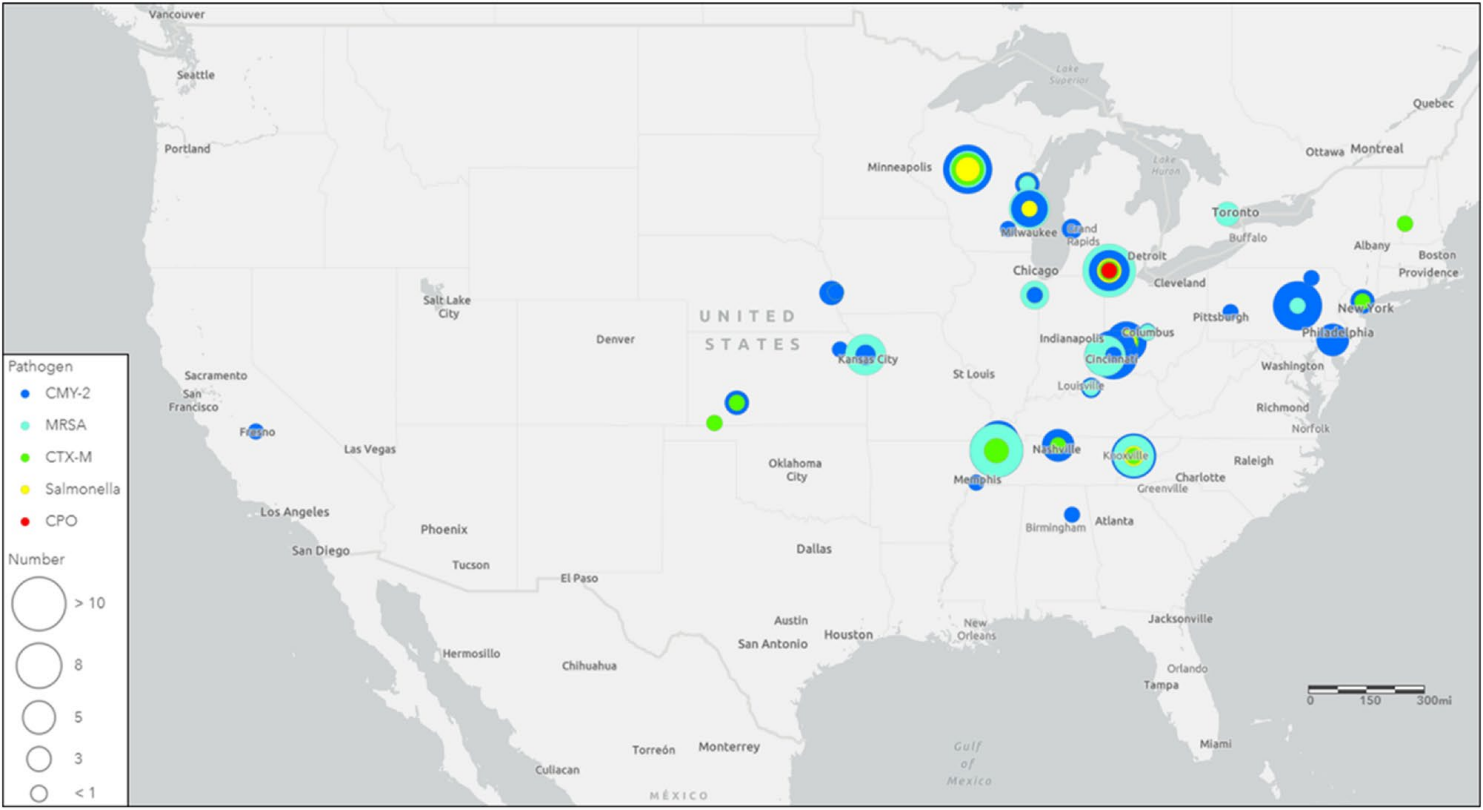
Map of meat processing plants that had at least one bacterial contaminant isolated from a meat package processed at that plant. The size of the circle represents the quantitative number of meat packages that were contaminated with each of the individual meat pathogens. Only the isolate producing epidemic carbapenemase enzymes encoded by *bla*_KPC_ and *bla*_NDM_ was included as a CPO. This map was created using the “Light Gray Canvas” basemap from ArcGIS Online software provided by Esri.

Meat packages were purchased from 17 different grocery store locations representing six grocery retail chains located in central and western Ohio. Each grocery store location was visited between one to three times for a total of 29 different store visits. No differences in meat package contamination were observed between grocery chains except for *Enterobacteriaceae* harboring *bla*_CMY-2_ (*P* = 0.01) (Fig. 2). Purchased meat products were produced at 116 different meat-processing facilities distributed throughout the US and Canada that processed between 1 and 48 of the packages collected for this study. The highest frequency of isolate recovery was from packages of meat produced at processing plants located in the Midwest and Eastern US (Fig. 3).

### MRSA contamination and SCCmec typing

**Figure 4:**
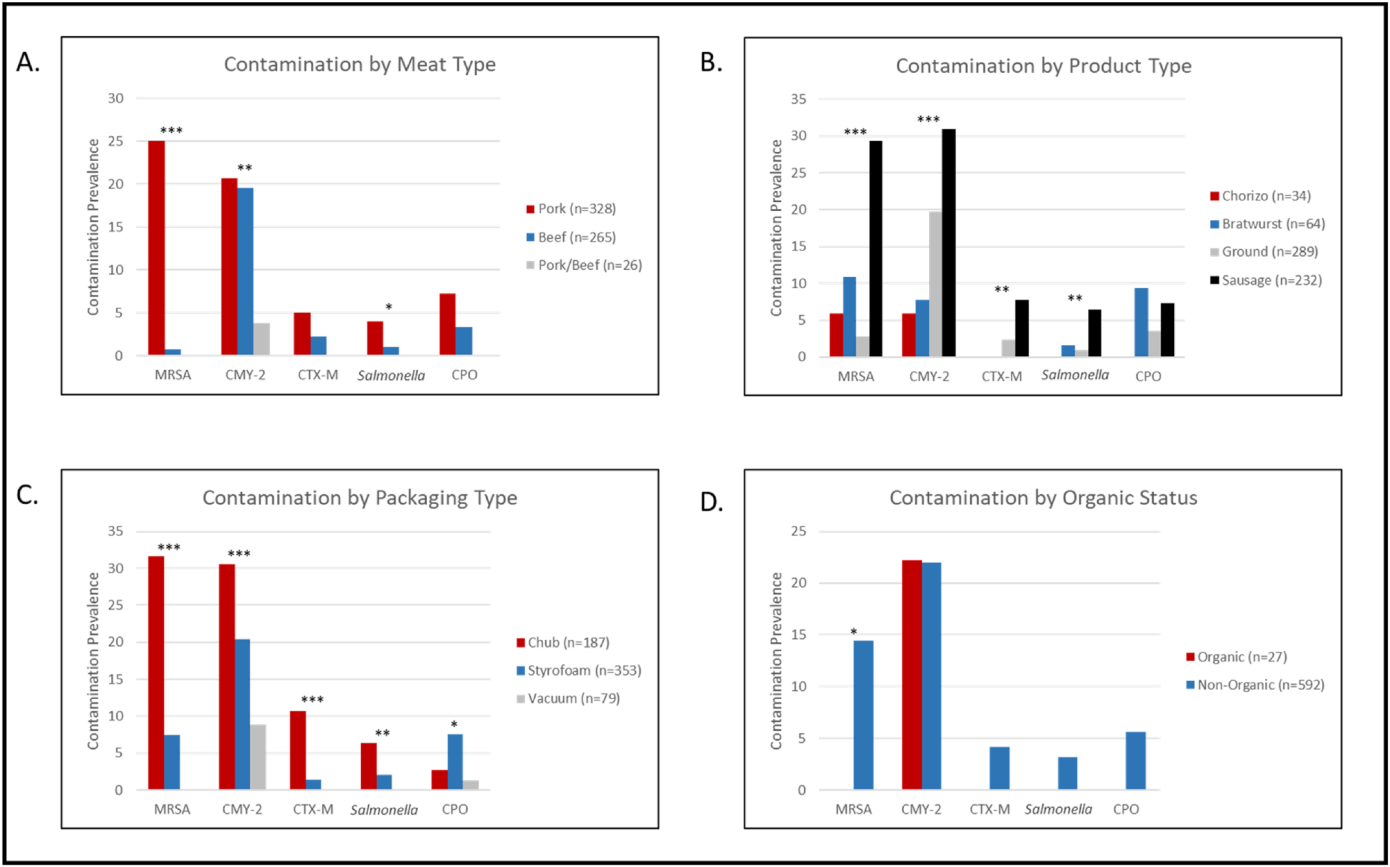
The contamination prevalence of each of the investigated contaminants by epidemiologic factors collected in this study: (**A**) Bacterial contamination by meat type; (**B**) Bacterial contamination by product type; (**C**) Bacterial contamination by meat packaging; (**D**) Bacterial contamination by organic status. Asterisks *P*-values for the Fisher exact test for association as follows: * = 0.05 > *P* > 0.01, ** = 0.01 ≥ *P* > 0.001; *** = 0.001 > *P*.

MRSA isolation was strongly associated with pork products (*P* < 0.001) (Fig. 4). In addition, the product type and packaging were associated with MRSA recovery (*P* < 0.001) with MRSA being more frequently recovered from sausage products and packaging that was not vacuum sealed. None of the 27 organic products were contaminated with MRSA, compared to 14.4% of the conventional products (*P* = 0.04). A total of 120 individual MRSA isolates were recovered from the 85 contaminated packages. SCCmec type IV was the most commonly isolated, contaminating 64 (75.3%) meat packages and accounting for 86 (72%) of the total MRSA isolates. SCCmec type VI and IVe were also identified, contaminating 13 (15.3%) and 6 (7.1%) meat packages and accounting for 17 (14.2%) and 9 (7.5%) of the isolates, respectively. An additional 8 (6.7%) isolates from 7 (8.2%) packages contained a mecA gene but could not be typed by multiplex PCR. Three pork sausage packages and two ground pork packages, harbored MRSA with two different SCCmec types.

### *Salmonella* contamination and characterization

Similar to MRSA, *Salmonella* was more frequently isolated from pork (4.0%) than from beef products (1.1%) or combined pork/beef (0%) products (*P* = 0.02) (Fig. 4). *Salmonella* were recovered more commonly from sausage products (15/232; 6.5%) compared to other product types (*P* = 0.004). While none of the vacuum-sealed products were contaminated with *Salmonella*, this pathogen was found in 12 (6.4%) and 7 (2.0%) chub and Styrofoam tray-packaged products, respectively (*P* = 0.005). Organic status was not associated with the recovery of *Salmonella*. Thirteen *Salmonella* serovars were identified among the 19 isolates. The most common serotypes were Montevideo and Agona accounting for 16% of the total *Salmonella* isolates each (Table 1). Twelve of the 19 isolates carried between 1 and 13 acquired antimicrobial resistance genes. The acquired cumulative resistome was quite diverse and frequently included fosfomycin (*fosA7*; 8/12), aminoglycoside (*aac, aph, aadA* alleles), sulfonamide (*sul1*), tetracycline, (*tet*) and extended-spectrum cephalosporin (*bla*_*CMY-2*_) resistance genes.

**Table 1:** *Salmonella* serovars isolated from 619 packages of fresh retail meat, stratified by meat type, product type, and packaging with acquired resistome data.

*S. enterica* serovar	Meat	Product	Packaging	Resistance genotype
Agona	Pork	Sausage	Chub	aac(3)-Via, aadA6, aph(3’’)-lb, aph(3’)-la, aph(6)-ld, blaCMY-2, floR, fosA7, qnrB19, rmtE1, sul1, sul2, tet(A)
	Pork	Sausage	Styrofoam	fosA7
	Beef	Ground	Styrofoam	fosA7
Montevideo	Pork	Sausage	Chub	aadA2, aph(3’)-la, fosA7
	Pork	Sausage	Chub	aac(6’)-laa, fosA7
	Ground	Beef	Styrofoam	fosA7
Anatum	Pork	Sausage	Chub	–
	Pork	Sausage	Chub	–
Albany	Pork	Sausage	Chub	–
Berta	Pork	Sausage	Chub	–
Derby	Pork	Sausage	Styrofoam	aadA2, fosA7, sul1, tet(A)
Dublin	Beef	Ground	Chub	aph(3’’)-lb, aph(6’)-ld, blaCMY-2, floR, sul2, tet(A)
Hindmarsh/Bovismorbificans	Pork	Bratwurst	Styrofoam	–
Newport	Pork	Sausage	Chub	–
Reading	Pork	Sausage	Styrofoam	fosA7
Saintpaul	Pork	Sausage	Styrofoam	–
Worthington	Pork	Sausage	Chub	blaCMY-2, tet(C)
Typhimurium strain TW-Stm6 chromosome	Pork	Sausage	Chub	tet(B)
Unidentified	Pork	Sausage	Chub	aac(2’)-lla, fosA7

### Recovery of *Enterobacteriaceae* expressing AmpC and ESBL genotypes

Isolates expressing *bla*_CMY-2_ (*P* = 0.007), and those expressing *bla*_CTX-M_ (*P* = 0.07) (Fig. 4) were most frequently isolated from pork products. Both the AmpC and ESBL genotypes were frequently isolated from sausage and ground products, and product type was associated with recovery of isolates expressing *bla*_CMY-2_ (*P* < 0.001) and *bla*_CTX-M_ (*P* = 0.004). Similar to MRSA and *Salmonella*, non-vacuum sealed packaging was associated with recovery of isolates expressing *bla*_CMY-2_ and *bla*_CTX-M_ (*P* < 0.001). The recovery of AmpC and ESBL *Enterobacteriaceae* was not associated with organic status.

The *bla*_CTX-M_-harboring *Enterobacteriaceae* were further characterized with regards to their allelic population structure. Among these 25 isolates, 4 harbored *bla*_CTX-M-1_ and 21 harbored *bla*_CTX-M-15_. *bla*_CTX-M-1_ isolates were recovered from Styrofoam tray-packaged ground beef as well as ground beef and pork chubs. Fifteen isolates with *bla*_CTX-M-15_ were recovered from ground pork chubs, two from ground beef chubs, two from ground pork in Styrofoam trays, and two from ground beef in Styrofoam trays.

### Carbapenemase-producing organism contamination and characterization

**Figure 5:**
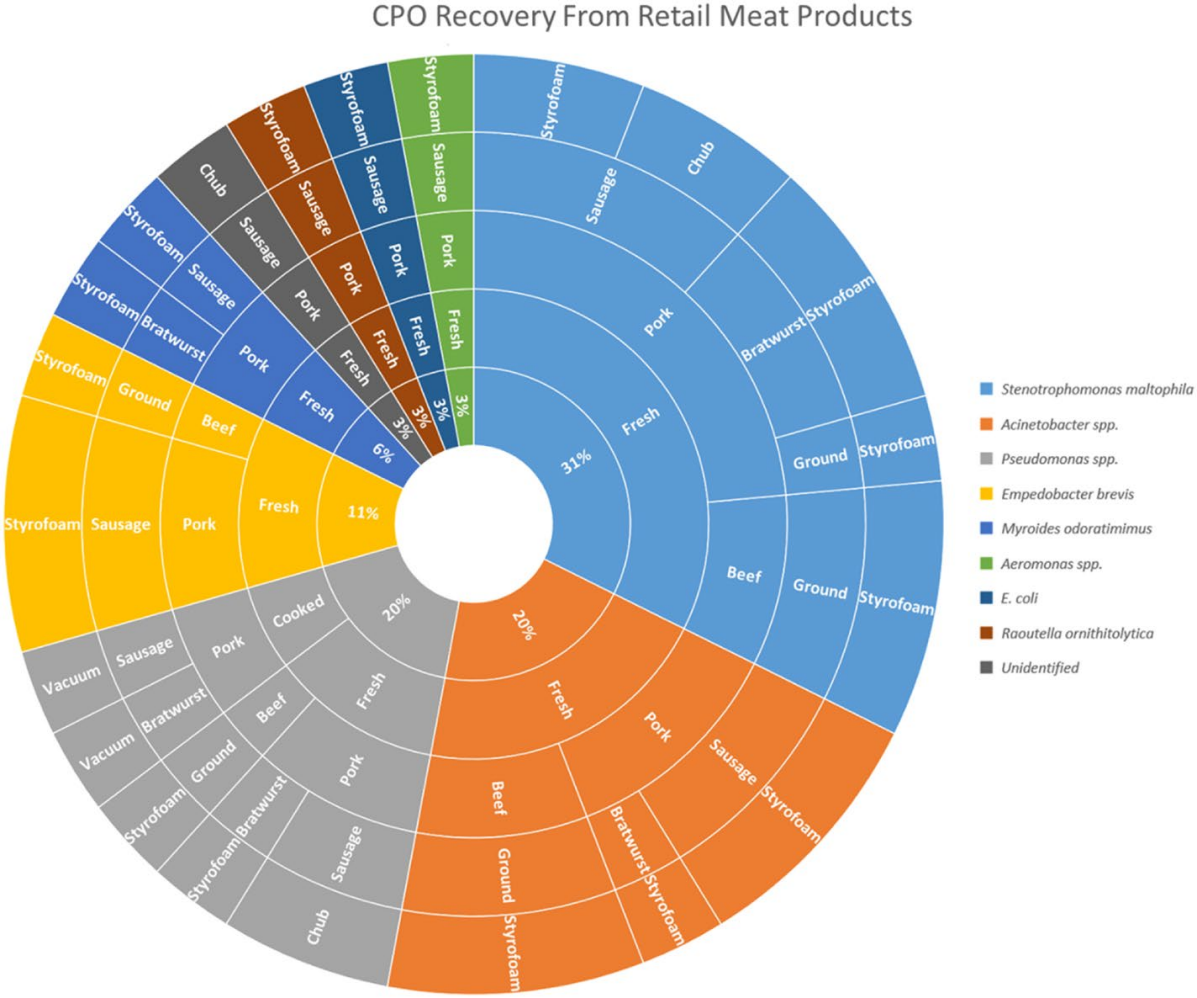
Hierarchal sunburst chart identifying the carbapenemase-producing organisms (CPO). The inner ring identifies the prevalence of each CPO species divided by the total number of CPO isolates. Outer rings further stratify each species by cooked status, meat type, product type, and meat packaging. Sunburst charts were visualized using Microsoft Excel graphing tools.

There was no association between CPO recovery and meat type, product type, or organic status. CPO recovery varied between meat packaging and was most prevalent among non-vacuum sealed Styrofoam tray packaging, followed by chub packaging and less frequently from vacuum-sealed products (*P* = 0.01). We isolated a diverse collection of carbapenemase-producing species that consisted primarily of *Stenotrophomonas maltophilia, Acinetobacter* spp., *Pseudomonas* spp. and *Empedobacter brevis* (Fig. 5). One *E. coli* was isolated from fresh pork sausage produced carbapenemase but did not harbor any of the investigated carbapenemase genes based on PCR.

**Figure 6:**
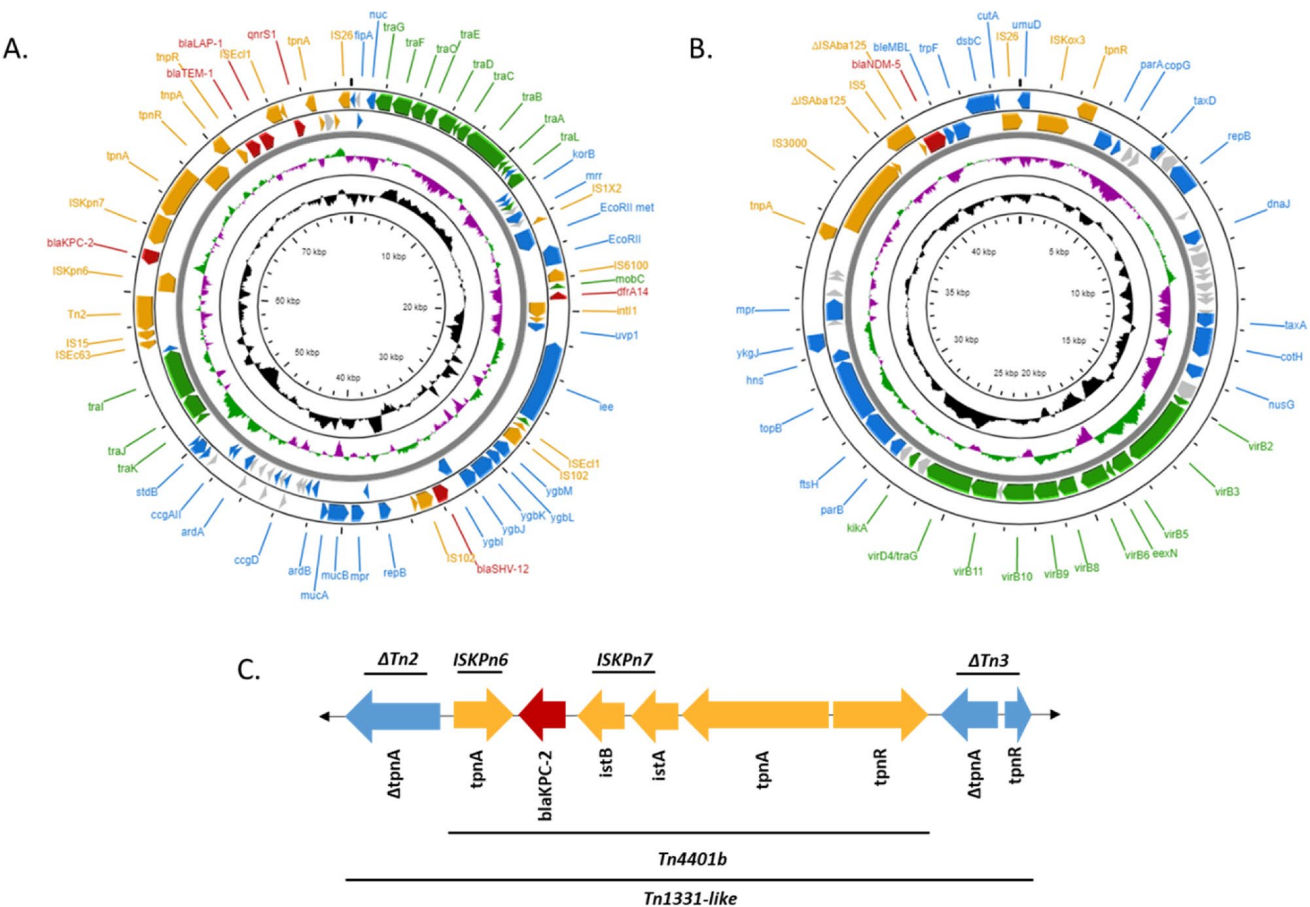
Plasmid annotations of the IncN (**A**) and IncX3 (**B**) plasmids containing the *bla*_KPC-2_ and *bla*_NDM-5_, respectively. Genes involved in conjugation, mobile genetic elements and antimicrobial resistance are depicted in green, orange and red, respectively. Other annotated genes and hypothetical genes are depicted in blue and gray, respectively. The nested transposon carrying the *bla*_KPC-2_ gene on the IncN plasmid is highlighted (**C**). The *bla*_KPC-2_ (red) is mobilized by the Tn4401b element (orange) nested within a truncated Tn1331-like element (blue). Annotation maps were visualized using CG Viewer.

We found that one CPO, a *Raoultella ornithinolytica* recovered from fresh pork sausage patties in Styrofoam tray packaging, harbored both *bla*_KPC-2_ and *bla*_NDM-5_. The isolate’s acquired resistome included *bla*_*LAP-1*_, *bla*_*SHV-12*_, *bla*_*TEM-1B*_, *qnrS1*, *fosA*, and *dfrA14* in addition to the acquired carbapenemase genes. The *bla*_KPC-2_ was harbored on a 78,869 bp IncN plasmid within a Tn4401b insertional element that inserted in a Tn1331-like element forming a nested insertional element (Fig. 6A,B). This plasmid shares significant sequence identity (Coverage 92%, Identity 99.77%) with an *E. coli* from a human bronchoalveolar lavage sample (Genbank ID: KJ933392). The *bla*_NDM-5_ was located on a 45,048 bp IncX3 plasmid within a complex insertional element consisting of an IS3000 and IS5 element upstream and IS26 transposase and ISKox3 element downstream (Fig. 6C). The intergenic region between the IS3000 and IS5 transposases and the IS5 transposase and *bla*_NDM-5_ contained truncated ISAba125 sequences. The IncX3 shared significant sequence identity (Coverage 100%, Identity 99.85%) with multiple plasmids derived from *E. coli, Klebsiella pneumoniae* and *Salmonella* Typhimurium isolated from humans, water and minced pork products (Genbank IDs: AP023210.1, CP024825.1, CP065346.1, CP053295).

Phenotypically, the isolate was multi-drug resistant expressing resistance to 20 different antimicrobials from seven different drug categories (Supplemental Table 1). However, it retained susceptibility to tetracyclines, aminoglycosides, chloramphenicol, and colistin. There was no zone of inhibition present when the isolate was grown in the presence of imipenem alone or when synergized with EDTA, suggesting that the KPC enzyme retains functionality and confers carbapenem resistance despite neutralization of the NDM by chelating the metallo-cofactor.

## Discussion

Of the 763 retail meat products purchased for this study, and we found that 29.4% (224/763) of these samples were contaminated with at least one of the targeted bacteria. Contamination was not associated with the retail grocery store chain from which the meat was purchased. Meat package contamination appeared to be clustered within products from meat processing plants located in the US Midwest. However, this is reflective of our sampling because most of the meat products came from Midwest processing plants. As expected, and previously reported, bacterial contamination was less frequently observed in cooked products because of their post-processing treatment^12,13^. Despite this, we still identified bacterial contamination by both CPO and *Enterobacter*iaceae harboring *bla*_CTX-M-15_ in cooked products. These CPO were *Pseudomonas* spp*.* that represent ubiquitous environmental isolates that typically have intrinsic, chromosomally encoded carbapenemase genes and did not harbor any of the transmissible, epidemiologically important CPO genotypes^14,15^.

Pork products were more frequently contaminated with MRSA, *Salmonella*, and *Enterobacteriaceae* producing CMY-2 and CTX-M than were beef or pork/beef combination products. Previous surveillance studies have reported similar trends when comparing MRSA, *Salmonella,* and *Enterobacteriaceae* producing CMY-2 between pork and beef^5,16,17^. Previous studies have identified swine as an important reservoir for MRSA and have reported that swine may have a higher on-farm prevalence of *Salmonella* compared to dairy and beef cattle operations^18,19^. While the microbial contamination we observed for beef products is similar to previous studies, we observed a higher contamination prevalence among these pork products compared to traditionally surveyed products like whole cuts and ground pork^5,8,20^. This could be due to the meat products we tested, as ground and processed pork products are almost exclusively from cull sows, while cuts are typically from younger finisher/market hogs. The lifespan of cull sows is typically longer than market hogs providing a greater opportunity to become colonized with resistant commensal and pathogenic bacteria that can contaminate the carcass during processing.

A number of risk factors were associated with our recovery of these targeted bacteria including the meat product and the type of packaging. Of all meat product types, sausage was the most frequently contaminated with nearly all bacterial contaminants except CPO. One study found that ground fresh pork and pork sausage in stores accounted for higher mean aerobic, coliform, and *E. coli* colony counts compared to whole cuts^21^. Non-vacuum packaging, both chubs and Styrofoam trays were more frequently contaminated than vacuum-sealed packaging. Vacuum sealed products create a nearly anaerobic environment that inhibits aerobic bacteria like MRSA and limits logarithmic growth of facultative anaerobes, creating a less contaminated space^22^. Like previous studies, organic status did not influence bacterial contamination except for MRSA^23^. The difference in MSRA prevalence between conventional and organic products may be a result of our small sample of organic products included in this study as others have reported similar rates of contamination between pork products from traditional and alternatively raised pigs^6^. Importantly, in our data meat type (pork vs. beef), product type, and packaging were highly correlated, and we could not control this confounding bias using multivariable models. For example, most of the processed pork products available for sampling at grocery stores in this study were sausage in non-vacuum sealed packages. This lack of sample variety made it difficult to discern true associations between contamination and package characteristics.

The MRSA recovered from the meat products in this study largely came from fresh pork sausage in chub or Styrofoam tray packaging. The majority of the isolates represented three SCCmec types, IV, IVe and VI. These SCCmec types are often attributed to community-acquired MRSA isolates and are believed to have enhanced fitness and the capacity for easier transmission^24^. However, SCCmec type IV MRSA have been reported from retail meat in the US^25^. Interestingly, the SCCmec types IVa, V, and X, often associated with MRSA from livestock and most commonly reported in retail meat, were not isolated from any of the meat products we collected for this study^26^. Nearly 7% of the MRSA isolates we recovered harbored a mecA gene but did not react to the multiplex PCR that includes SCCmec types I-VI. It is possible that these isolates could be unconventional SCCmec types that are often attributed to MRSA of animal origin^26^.

We isolated nontyphoidal *Salmonella* from 19 of the packages of fresh retail meat. The isolates were primarily recovered from pork sausage packaged in chubs. Diverse serotypes were observed, including 14 isolates representing 8 serotypes that are among the top 32 *Salmonella* serotypes reported to cause human disease in the US^27^. Half of the *Salmonella* isolates were pansusceptible, four had only one commonly acquired resistance gene, and six had more than one acquired resistant gene. The most common resistance genotypes were *fosA7* and aminoglycoside-resistance alleles (*aph*, *aadA* and *aac*), followed by *sul* and *tet* alleles and *bla*_CMY-2_. These acquired genes confer resistance to a number of medically important antibiotics used in both human and veterinary medicine including some of the most frequent antimicrobials sold for use in cattle and swine production^28^.

We identified 17.8% (136/619) and 4.0% (25/619) of fresh retail meat packages that were contaminated with *Enterobacteriaceae* expressing *bla*_CMY-2_ or *bla*_CTX-M_, respectively. This level of contamination with *Enterobacteriaceae* producing CMY-2 is similar to previous reports in pork and beef, as is the observation that pork products were more frequently contaminated than beef^5^. *Enterobacteriaceae* producing CTX-M are not widely reported in beef and pork products in the US, with only two samples—one from ground beef and one from pork chops—that harbored the CTX-M genotype over 6 years of national surveillance^7^. Our findings suggest that CTX-M contamination is more prevalent in products not typically sampled in national surveillance including ground pork and ground pork sausage products. Infections with ESBL-producing *Enterobacteriaceae* increased nearly 50% since 2012 and are considered a serious public health threat by the CDC^29^. Although direct zoonotic foodborne transmission of *Enterobacteriaceae* producing CMY and CTX-M is not commonly reported, circumstantial evidence suggests that retail meat might contribute to a portion of the nearly 93,000 estimated community-acquired cephalosporin-resistant infections in the US each year^30–32^. Most of our ESBL-producing isolates harbored *bla*_CTX-M-15_ alleles, a variant that is widely disseminated among the human population and causes widespread infections^33^.

CPO were recovered from multiple samples and included a variety of bacterial species. These included soil- and water-associated organisms that are often considered intrinsically resistant and/or maintain carbapenemase genes that are not mobilized^14,15^. Although these species can cause documented clinical infections, only one package harbored a carbapenemase-producing bacteria with epidemic, transferrable carbapenemase genes. One package of fresh pork sausage was contaminated with a *R. ornithinolytica* that harbored two epidemic carbapenemase genes, *bla*_KPC-2_ and *bla*_NDM-5_. Epidemic carbapenemase genes in *Enterobacteriaceae* have been identified from sow fecal samples and their environments^10,34^. However, the carbapenemase genotypes identified in those studies were *bla*_IMP_ alleles. Functional and transferable *bla*_KPC_ has been identified in the cattle fecal microbiome, but bacterial organisms harboring these genotypes—*bla*_KPC_ and *bla*_NDM_—have not been reported from livestock in the US^11^. Comparatively, these epidemic carbapenemase genes are commonly reported in bacteria recovered from humans, wastewater, and environmental sources like surface water^35–37^. Post-harvest processing and packaging is a potential source for meat contamination with CPO and may serve as a reservoir for human and environmentally associated genotypes to enter the food chain^38,39^. Our data indicate that bacteria expressing epidemic carbapenemase genes can enter the food supply and represent a new foodborne transmission pathway contributing to the emerging CPO public health threat.

Mobilization of carbapenemase genes is a leading factor for their widespread dissemination beyond human hospitals to communities, natural environments, and domestic and wild animals. The epidemiologic dissemination of *bla*_NDM-5_ through members of Enterobacterales is partially a function of the self-transmissible IncX3 plasmid that drives their spread within and beyond hospitals into the community^40,41^. Moreover, the insertional element in our *bla*_NDM-5_ shared similarity to a number of insertional elements carrying *bla*_NMD-5_ and *bla*_NDM-1_ with full or truncated IS3000 and ISAb125 insertion elements^41,42^. This supports the hypothesis that the NDM enzyme mobilized from the chromosome of *Acinetobacter* spp. and spread to Enterobacterales. Our *bla*_KPC-2_ is on the Tn4401b transposon, an isoform of the Tn4401 transposable element that is responsible for epidemic expansion and dissemination of the *bla*_KPC-2_^43^. The unique nature of the nested transposon reported here has been previously observed in a clinical isolate from the US^44^. These elements are widely disseminated to plasmids, including the IncN, that facilitate global dissemination of *bla*_KPC-2_ among Enterobacterales^45^.

The presence of both *bla*_KPC-2_ and *bla*_NDM-5_ in same isolate from an environmental source seems an exceptionally rare event. Simultaneous maintenance of other metallo-β-lactamases with a KPC serine carbapenemase is recognized as an emerging antimicrobial resistant threat^46,47^. Co-harborization of *bla*_KPC_ and *bla*_NDM_ is recognized internationally, mainly in clinical case reports from hospital settings where genotype combinations consist of NDM-1 or NDM-5 paired with KPC-2^47–49^. Phenotypic analysis using an EDTA synergy test identified that the KPC maintained functional carbapenem-resistance when the NDM was neutralized by chelation of the enzymatic cofactor. This is an important finding since limited conjugation-expression studies from previously reported clinical isolates suggest the NDM confers a higher resistance breakpoint compared to the KPC, although not in every case^47^. Nonetheless, co-harborization offers a more complete and comprehensive resistance to carbapenems and carbapenem/β-lactamase inhibitor combinations^47–49^.

This study has some limitations that should be considered when interpreting the results. Our phenotypic screening methods to recover CPO that utilized carbaNP testing and PCR genotyping may have missed some CPO genotypes harboring allelic variants of *bla*_OXA-48_. However, these screening methods have been successfully used to identify bacteria expressing clinically-relevant carbapenem-resistance phenotypes and CPO genotypes causing epidemic infections or that are disseminated in the environment and livestock in the US^10,34,36,37^. Isolates unable to grow at carbapenem concentrations below what is considered clinically resistant or in the presence of cephalosporins are unlikely to be considered an important public health threat. In addition, while our method for phenotypically screening MRSA using oxacillin is frequently used in *S. aureus* surveillance studies, it may underestimate the true prevalence of MRSA when compared to other methods such as cefoxitin disc diffusion due to variablity in expression of the mecA gene. Moreover, we did not test for the mecC gene as part of our genotypic screening of MRSA suspect isolates. Despite this, our results identify the prevalence of the mecA among all phenotypically resistant MRSA and suggest a preponderance of these human-associated strains contaminating retail meat products.

Here we show that fresh retail pork and beef products, including raw, processed meat, pose a similar public health risk for the foodborne transmission of pathogens and antimicrobial resistant bacteria as other meat products that are more frequently included in national surveillance programs. Moreover, we identified a *R. ornithinolytica* from fresh pork sausage that harbors multiple carbapenemase genes on mobile plasmids. This finding is concerning and suggest that clinically important CPO have the potential to enter the food supply through zoonotic transmission or post-processing contamination. Our results suggests that a more diverse range of meat products, including raw processed meats should be included in national surveillance programs as they serve as vehicles of antimicrobial resistant foodborne pathogens.

## Methods

### Sample collection

We purchased beef and pork products available for retail sale from a convenience sample of grocery stores located in central and western Ohio over a period of 16 weeks during the summer of 2018. Grocery stores included both large chain stores and local stores with a variety of fresh meat products available for retail sale. We purchased retail meat products from up to three different grocery stores per week and did not purchase meat products from the same grocery store more than once in a 2-week period. We purchased a single package of each unique ground beef or pork product of each available brand at each grocery store at a single visit. This sampling was intended to capture all available varieties of ground beef and pork products including both fully cooked and fresh products, as well as both seasoned and unseasoned. In individual stores where a large number of unique products were available, we purchased no more than 30 total meat products.

Meat products purchased for this study included ground beef, ground pork, ground breakfast sausage, specialty sausages (including bratwurst, chorizo, and kielbasa), meatloaf, and meatballs. We attempted to purchase both fresh and fully cooked products made from either beef or pork that were available in store wrapped Styrofoam trays, chubs, and modified atmosphere/vacuum packaging. For each product purchased we recorded the store and purchase date, as well as details of label information including seasoning, organic status, sell-by date, and processing plant identification number.

### Culture and identification of methicillin-resistant *Staphylococcus aureus*

A 10 g aliquot of each meat sample was sterilely inoculated into 90 mL of trypticase soy broth (TSB; Becton Dickinson, Sparkes, MD) supplemented with 2% salt, incubated overnight at 35 °C, and inoculate onto Mannitol Salt agar (Becton Dickinson) for identification of MRSA suspect isolates. Up to three different morphologies were selected from the Mannitol salt agar and sub-cultured onto tryptic soy agar with sheep’s blood. Hemolysis was assessed and all morphologies were tested for coagulase production. We then inoculated coagulase positive *Staphylococcus* suspects onto oxacillin-screening agar to identify methicillin resistant strains. Methicillin-resistant *Staphylococcus spp.* were speciated by conventional multiplex PCR of the *nuc* gene^50^. We simultaneously tested phenotypically methicillin-resistant *S. aureus* strains for the presence of the *mecA* gene and typed their staphylococcal chromosomal cassette *mec* (SCCmec) using a previously described multiplex PCR and by comparing gel electrophoresis banding patterns to known controls^51^.

### Culture and identification of *Salmonella* spp.

A second 10 g aliquot from each package of meat was sterilely inoculated into 90 mL of buffered peptone water (BPW; Becton Dickinson) and incubated overnight at 37 °C. We transferred a 100 µL aliquot of the meat/BPW homogenate to 10 mL of Rappaport-Vassilidis R10 (RV; Becton Dickinson) broth that was then incubated overnight at 42 °C. RV broth was subsequently inoculated onto xylose-lysine-Tergitol 4 agar (XLT-4; Becton Dickinson) for the differentiation of *Salmonella* suspect isolates. Characteristic black colonies on XLT-4 agar were tested for lactose fermentation on MacConkey agar and agglutination with polyvalent antisera O. Lactose negative, agglutinating isolates were speciated using MALDI-TOF, and confirmed *Salmonella* underwent whole genome sequencing (MiSeq, Illumina, San Diego, CA). Reads were assembled using the SPades assembler version 3.9 and post-processed with MisMatch corrector available online from the Center of Genomic Epidemiology (CGE)^52^. We confirmed *Salmonella* genus and species, identified *Salmonella* serotype, and determined acquired antimicrobial resistance genotype using the KmerFinder, SeqSero, and ResFinder online databases available at CGE^53–55^.

### Culture and identification of extended-spectrum cephalosporin-resistant *Enterobacteriaceae* and carbapenemase-producing organisms

A 10 g aliquot from each package of meat was sterilely inoculated into 90 mL of MacConkey broth (Becton Dickinson) modified with 2 µg/mL cefotaxime and incubated overnight at 37 °C. An inoculate of the MacConkey broth was then streaked onto three MacConkey agar plates that were modified with either 8 µg/mL of cefoxitin, 4 µg/mL of cefepime, or 0.5 µg/mL meropenem and 70 µg/mL zinc sulfate heptahydrate for the isolation of Enterobacterales expressing the AmpC beta-lactamase-producing phenotype, ESBL-producing phenotype, or carbapenemase-producing phenotype, respectively. We have previously used this method to successfully recover isolates representing the intended genotypes^5,10,23,34,37^.

We selected a single lactose-fermenting isolate expressing the AmpC beta-lactamase-producing phenotype and another expressing the ESBL-producing phenotype for further characterization. These isolates were tested for tryptophan utilization using the indole production assay. Isolates were then genotypically characterized for the presence of either *bla*_CMY_ or *bla*_CTX-M_ by PCR using previously reported primers^56,57^. PCR products of the expected molecular weight were cleaned and bidirectionally sequenced using a 3730 DNA analyzer (Applied Biosystems) and analyzed for allelic variation using the basic local alignment search tool (BLAST).

We also selected up to three morphologically distinct colonies with a carbapenemase-producing phenotype, giving preference to lactose fermenting isolates. We confirmed carbapenemase production using the CarbaNP test and then determined bacterial genus and species using MALDI-TOF^58^. Isolates representing bacterial species not expected to have intrinsic carbapenem-resistance—*E. coli, Klebsiella, Enterobacter, Proteus, Raoultella, Shewenella, Morganella, Providencia, Acinetobacter* and *Pseudomonas aeruginosa*—were tested for the presence of *bla*_KPC_, *bla*_NDM,_ *bla*_IMP_ and *bla*_VIM_ by PCR using previously reported primers^59–62^.

An isolate with transmissible carbapenemase genes underwent whole genome sequencing, using both short-read (MiSeq, Illumina, San Diego, CA) and long-read (PacBio; Pacific Biosciences, Menlo Park, CA) platforms. Adapter sequences were trimmed, and sequencing data were quality assessed using TrimGalore and FastQC, respectively^63,64^. The resulting sequences were assembled using Unicycler to generate a hybrid assembly^65^. Assembled sequences were assessed for acquired antimicrobial resistance genes and plasmid content using ResFinder and PlasmidFinder, respectively^55,66^. The resulting contigs were also annotated using Prokka and plasmid sequences were further annotated using BLAST^67^. Insertion sequences and integrons were characterized using ISFinder and INTEGRALL and plasmid gene maps were viewed using CGViewer^68–70^.

An antimicrobial susceptibility profile was also generated for the isolate with transmissible carbapenemase genes using the Sensititre semi-automated broth micro-dilution system (NARMS CMV3AGNF, ESB1F and GNX2F panels; Thermo Fisher Scientific, Oakwood Village, OH) following Clinical and Laboratory Standards Institute (CLSI) guidelines^71^. This isolate was also subjected to the EDTA-disk synergy test to determine and differentiate serine carbapenemase and metallo-carbapenemase production^72^.

### Data summarization and analysis

Prevalence data of individual foodborne bacteria were stratified and summarized by individual predictor variables. We used Fisher’s exact test to determine the unadjusted association between the recovery of the individual types of bacteria and each of the individual predictors. Multivariable models were considered for data analysis, but the correlation of predictor variables resulted in collinearity. Analysis of associations for all predictors except meat type were conducted only on data from fresh retail meat samples as the prevalence of foodborne bacteria was negligible in cooked products. Statistical analyses were conducted using STATA v15.1 (StataCorp LLC, College Station, TX, USA) and heatmaps were visualized using HeatMapper^73,74^. The map used in this manuscript was created using ArcGIS® software by Esri^75^. ArcGIS® and ArcMap™ are the intellectual property of Esri and are used herein under license. Copyright © Esri. All rights reserved. For more information about Esri® software, please visit www.esri.com.

### Genomic data

Whole genome sequencing data of *R. ornithinolytica* chromosomal and plasmid DNA were deposited in GenBank under the accession numbers CPO54270-CPO54276. Whole genome sequences of *Salmonella* isolates were submitted to GenBank via the GenomeTrakr network and can be found under the bioproject PRJNA338674 as biosamples SAMN09850703, SAMN09850714, SAMN09850522, SAMN09850520, SAMN09850343, SAMN09850452, SAMN09850300, SAMN09850301, SAMN09850841, SAMN09850839, SAMN09850833, SAMN09851034, SAMN09850707, SAMN09850838, SAMN09850710, SAMN10883380, SAMN10883352, SAMN10883383, and SAMN10814272.

## Supplementary Information

Supplementary Table.
