# Supplementary material for: Antimicrobial resistant bacteria recovered from retail ground meat products in the US include a Raoultella ornithinolytica co-harboring blaKPC-2 and blaNDM-5
Source: Sci Rep. 2021 Jul 7;11:14041. doi: 10.1038/s41598-021-93362-x (PMC8263791; doi:10.1038/s41598-021-93362-x)
Supplement: Supplementary file 1 — Supplementary Table. [file 41598_2021_93362_MOESM1_ESM.docx]

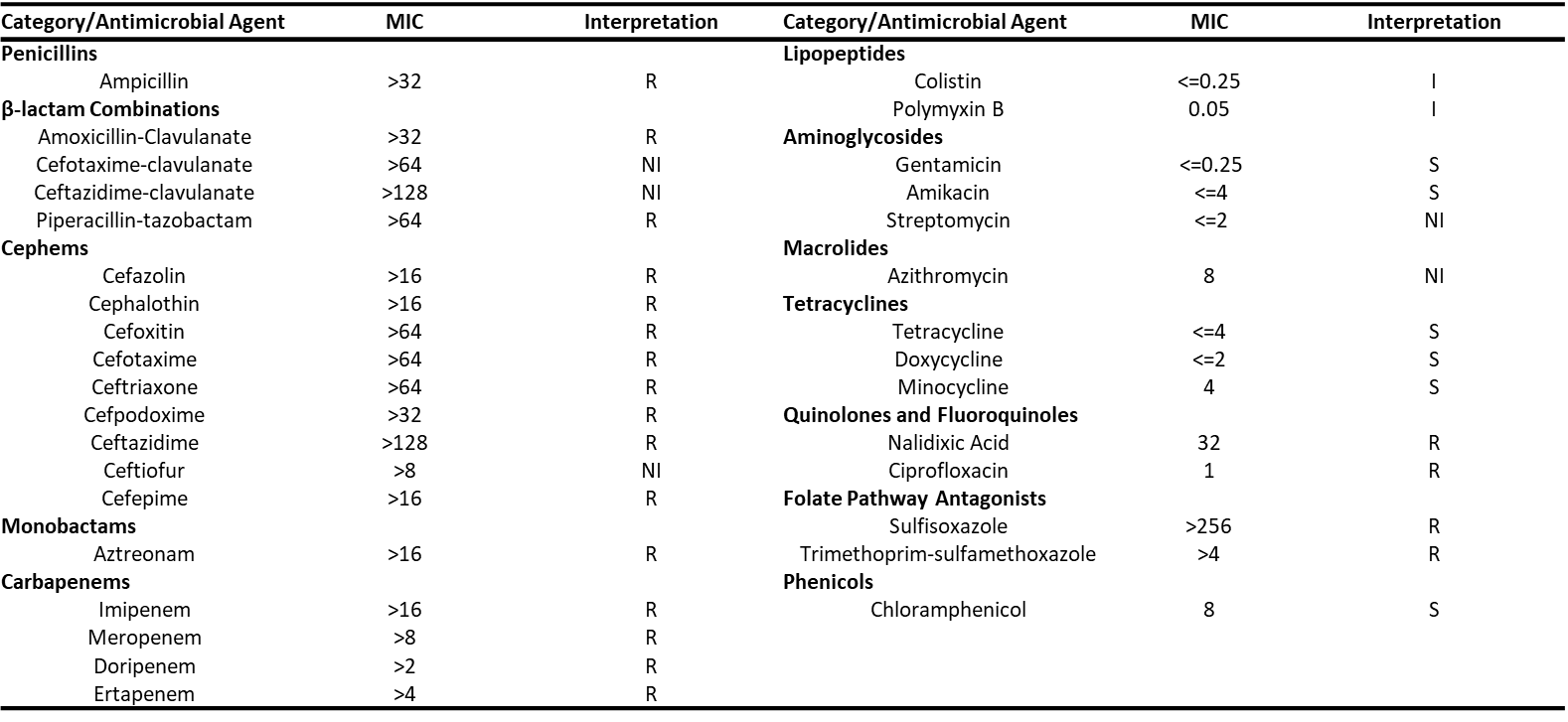


Supplemental Table 1. The antimicrobial minimal inhibitory concentration breakpoint values and their interpretations for the *Raoultella ornithinolytica* harboring both *bla*_KPC-2_ and *bla*_NDM-5_. Abbreviations: R=resistant, I=Intermediate, S=susceptible, NI=no interpretation for the given antimicrobial tested.
